# Meta-Analysis of Randomised Clinical Trials Comparing Idarubicin + Cytarabine with Daunorubicin + Cytarabine as the Induction Chemotherapy in Patients with Newly Diagnosed Acute Myeloid Leukaemia

**DOI:** 10.1371/journal.pone.0060699

**Published:** 2013-04-05

**Authors:** Jing Wang, Yong-Gong Yang, Min Zhou, Jing-Yan Xu, Qi-Guo Zhang, Rong-Fu Zhou, Bing Chen, Jian Ouyang

**Affiliations:** Department of Hematology, the Affiliated DrumTower Hospital of Nanjing University Medical School, Nanjing, Jiangsu, PR China; Cardiff University, United Kingdom

## Abstract

**Background:**

To determine whether the use of idarubicin+cytarabine (IA) is more effective than the use of daunorubicin+cytarabine (DA) as induction chemotherapy for patients with newly diagnosed acute myeloid leukaemia.

**Methods:**

A computer-based search was performed. Randomised trials comparing IA with DA as induction therapy for newly diagnosed AML were included in this meta-analysis. The primary outcome of interest for our analysis was survival (disease-free survival, event-free survival and overall survival); the secondary endpoint was complete remission.

**Results:**

Ten trials with 4,060 patients were eligible for this meta-analysis. Our pooled results suggest that IA is associated with a significant advantage in CR (RR = 1·23; 95% CI = 1·07–1·41, *p* = 0.004), EFS (HR = 0·64; 95% CI = 0·45–0·91, *p* = 0.013), and OS (HR = 0·88; 95% CI = 0·81–0·95, *p* = 0.02) but not in DFS (HR = 0·90; 95% CI = 0·80–1·00, *p* = 0.06). In the subgroup analysis, age had a significant interaction with OS and CR benefits.

**Conclusion:**

Our analysis indicated that IA could improve the duration of overall survival compared to DA as induction therapy for young patients with newly diagnosed AML. Further study is needed to determine whether IA can produce clinical benefits in selected genetic or molecular subgroups of young AML patients.

## Introduction

Acute myeloid leukaemia (AML) is an extremely heterogeneous malignant disease resulting from acquired mutations that block the differentiation of primitive haematopoietic cells, thereby causing immature myeloid precursors to accumulate, resulting in an estimated 13,330 cases and an estimated 8,950 deaths in the United States in 2010 [1]. As of today, the management of AML remains a challenge for haematologists. The first goal of treatment is to achieve complete remission (CR), and further treatment is performed to prevent relapse. Much focus has been placed on increasing CR and reducing relapse and mortality to increase disease-free-survival (DFS), event-free survival (EFS), and overall survival (OS). Several trials have suggested the potential utility of cladribine or gemtuznmab-ozogamycin for remission induction therapy [2]–[5]; however, the “3+7” protocol currently remains the standard remission induction therapy for AML. The current recommendation for young AML patients from the National Comprehensive Cancer Network (NCCN), based on a literature review and on expert consensus, is three days of an anthracycline (e.g., daunorubicin at a dose of at least 60 mg/m^2^ or idarubicin at a dose of 12 mg/m^2^), and seven days of cytarabine (100–200 mg/m^2^ continuous infusion). For patients younger than 60 years old, the induction therapy generally consists of 3 days of an anthracycline (e.g., daunorubicin at 45–60 mg/m^2^ or, as an alternative, idarubicin at 12 mg/m^2^) and 7 days of cytarabine (100–200 mg/m^2^ continuous infusion) (V2·2011: available at http://www.nccn.org). The European Leukaemia Net (ELN) [6] also provides similar recommendations for AML treatment. These recommendations suggest that the choice of an anthracycline (daunorubicin or idarubicin) is of little consequence, assuming that equipotent doses are administered.

Daunorubicin is the first and most widely used anthracycline in remission induction therapy for AML. Many randomised trials, performed at several institutions across the world, have compared idarubicin with daunorubicin over the past two decades. In the 1990s, several randomised studies reported a prolonged survival effect of idarubicin, compared to daunorubicin, in combination with Ara-C [7], [8]. An IPD-based meta-analysis of five randomised trials comparing idarubicin with daunorubicin found that among patients achieving CR, fewer patients receiving idarubicin experienced relapses (*P* = 0·008), but somewhat more died during CR (*p* = 0·007), resulting in no significant DFS benefit [9]. Furthermore, OS improved with idarubicin compared with daunorubicin, with 13% versus 9% of patients, respectively, alive at 5 years (*P* = 0·03) [9]. However, because the durations of neutropenia and thrombocytopenia were longer in the idarubicin groups, whether the doses of anthracyclines used in these studies were equivalent in terms of the level of toxicity and whether any observed advantages represented an inherent biological advantage of idarubicin, rather than biological dose equivalence, were frequently questioned [10], [11]. The results of the meta-analysis were finally ignored. Finally, a total dose of more than 180 mg/m^2^ of daunorubicin was administered during the course of induction therapy to compare daunorubicin to idarubicin in recent randomised studies [12], [13], which was more than the standard dose of 40 to 50 mg/m^2^ given for 3 days. Interestingly, these comparative studies did not reveal survival differences in outcomes in patients between comparative regimens of cytarabine plus daunorubicin at a high dose (>180 mg/m^2^) or idarubicin at 36 or 48 mg/m^2^, suggesting therapeutic equivalence between the two drugs at these doses.

Which is the optimal anthracycline to use in AML, daunorubicin or idarubicin? There have been many studies aimed at establishing an ideal induction therapy for AML, but most of them have failed to demonstrate the true superiority of IA over DA. On-going randomisation between DA and IA is being administered to demonstrate whether the choice of an anthracycline is appropriate according to the recommendations of the NCCN and ELN regarding induction regimen (NCT01145846: available at http://www.clinicaltrials.gov). When used to compare results from different studies, a meta-analysis can test hypotheses about sources of differences and can assess the magnitudes of biases [14]. To obtain comprehensive estimates of the clinical benefit from all of the available data, we performed a meta-analysis of all of the relevant randomised trials that compared IA with DA in patients with newly diagnosed AML. This meta-analysis was performed in accordance with the Preferred Reporting Items for Systematic Reviews and Meta-Analyses (PRISMA) guidelines [15].

## Methods

### Search strategy

A computer-based search was performed of MEDLINE, EMBASE, the Cochrane-controlled trials registry, the Cochrane Library, and the Science Citation Index through March 2012. The search strategy included the medical subject headings of “Acute myeloid leukemia”, “idarubicin”, “daunorubicin”, and “anthracycline.” The reference lists were screened of all of the identified trials and of the comprehensive reviews in the field. The volumes of abstracts of the annual meetings of the American Society of Hematology (ASH), the European Haematology Association (EHA), and the American Society of Oncology (ASCO) were screened from 1995 to 2011. Prospective and on-going trials were identified by searching the following prospective trials registers: http://www.anzctr.org.au, http://www.clinicaltrials.gov, http://isrctn.org, http://www.trialregister.nl/trialreg/index.asp, http://www.umin.ac.jp/ctr.

### Inclusion and exclusion criteria

For inclusion, the trials had to be prospective and randomised, with IA chemotherapy in one arm compared with DA chemotherapy in the other arm as the induction therapy for patients with newly diagnosed AML. If the same author reported results that were obtained from the same patient population in more than one publication, then only the most recent or most complete report was included in the analysis. Trials including other chemotherapy drugs (e.g., etoposide) in their induction schedules were excluded because their induction regimens were different from the guidelines of the NCCN and ELN.

### Extraction process

A structured form was used to extract the relevant data from the trials. This extraction was performed independently by two reviewers. For studies including comparisons of different doses of idarubicin or daunorubicin, the data were extracted separately for each comparison group whenever possible. All data were checked for internal consistency, and disagreements were resolved by discussion among the investigators. The reviewers were not blinded to availability, as the abstracts were obtained personally.

### Methodological quality assessment

Quality assessment was based on the reporting of the study methods and results, namely randomisation, generation and concealment of treatment allocation, blinding, handling of withdrawals and dropouts, analysis by intention to treat, comparability of characteristics at baseline, treatment protocol being clearly described, outcome definition, and the extent of follow-up being clearly described. Study quality was coded as A (low risk of bias), B1 (low-moderate risk of bias), B2 (moderate-high risk of bias), or C (high risk of bias); as Liddle *et al.* commented [16], these codes are intended to be compatible with those of the Cochrane Collaboration (Cochrane Handbook, version 5.0.1, available at http://www.cochrane-handbook.org). We did not explicitly score the methodological quality of the included trials because the ad hoc quality assessment scores might have lacked demonstrated validity and the results might not have been associated with quality [14], [17].

### Definition of outcome

The primary outcome of interest for our analysis was survival (disease-free survival, event-free survival, and overall survival); the secondary endpoint was complete remission. The above information was extracted from each study. We did not define any minimum number of patients as a criterion for including a study in our meta-analysis.

### Statistical analysis

To estimate the treatment effects, the outcomes were calculated as either relative risks (RRs) or hazard ratios (HRs), with their respective 95% confidence intervals (CIs) (a benefit of IA would be represented by an HR<1 or RR>1). The survival outcome data were synthesised using the time-to-event HR as the effect measurement, and the other data were synthesised using the RR as the effect measurement. When HRs were not given in a paper, the data were extracted from the appropriate Kaplan-Meier curves, or the survival rates of each group were used to calculate the HRs [18], [19]. Heterogeneity assumptions were checked using the chi-square-based Q-test [20]. Heterogeneity was considered statistically significant if P<0.10, and it was quantified using the I^2^ metric, which is independent of the number of studies in the meta-analysis (I^2^<25%, no heterogeneity; I^2^ = 25–50%, moderate heterogeneity; and I^2^>50%, large or extreme heterogeneity). The random effects model adjusts for the variability of results among trials and provides a more conservative estimate of an effect using a wider CI [21]. However, a random effects analysis will give more weight to smaller trials, which it appears overestimate the benefit of treatment, leading to biased overall results [22]. Therefore, the pooled RR/HR estimate of each study was calculated by both the fixed-effects model (the Mantel–Haenszel method) [23] and the random-effects model (the DerSimonian and Laird method) [24]. In meta-analyses with at least four trials, Begg's test [25] and Egger's test [26] were performed to determine whether there was a publication bias (P<0.05 indicated a statistically significant publication bias). Moreover, contour-enhanced funnel plotting was performed to aid in interpreting the funnel plot [27].

One-way sensitivity analysis was performed to assess the stability of the results; specifically, a single study involved in the meta-analysis was deleted each time to reflect the influence of the individual data set on the pooled RRs/HRs. A subgroup analysis was conducted in an effort to determine whether modification of the inclusion criteria of this meta-analysis affected the final results. We performed the subgroup analysis, which was pre-planned according to the prepared protocol for this meta-analysis, by limiting the meta-analysis to studies using the following criteria: (a) time of publication, before or after 2003; (b) median age, older or younger than 60 years old; and (c) total dose of DNR, greater than or less than 180 mg/m^2^. Interaction tests were used to compare the differences between estimates from different subgroups [28]. All of our meta-analyses of efficacy outcomes were performed according to the intention-to-treat (ITT) principle. Review Manager (version 5·0 for Windows) and STATA, version 10.0, were used for the statistical analysis. The statistical tests for heterogeneity were one-sided, and the statistical tests for effect estimates and for publication bias were two-sided.

## Results

### Description of trials

**Figure 1:**
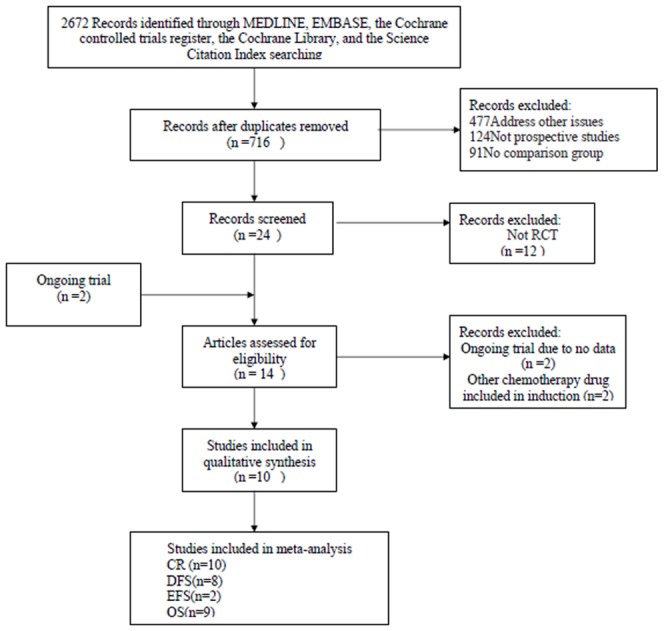
Process of identifying and selecting the relevant randomised controlled trials according to the PRISMA statement.

The process for the identification and selection of the relevant randomised, controlled trials (RCT), according to the PRISMA statement, is depicted in Figure 1. Since the 1990s, a total of 10 randomised trials have been described comparing IA and DA in newly diagnosed AML [7], [8], [12], [13], [29]–[34]. Two trials including etoposide in the induction schedule were excluded [35], [36]. The trials that fulfilled the inclusion criteria were conducted between 1984 and 2006, were published between 1991 and 2011, and included 4,060 patients (2,107 patients randomised to treat with IA and 1,953 control patients). Despite the three different comparative regimens of cytarabine plus daunorubicin at 80 mg/m^2^ for 3 days or idarubicin at 12 mg/m^2^ for 3 or 4 days, the study by Pautas *et al.* [12] was considered to be one individual comparison study (daunorubicin *vs.* idarubicin), according to the recommendations of the Cochrane Handbook, version 5.0.1.

All of the included trials were available as fully published papers. The characteristics of the trials included are shown in Table 1. CRs were reported in all of the studies. Survival data could be extracted from nine studies for OS [7], [8], [12], [13], [29]–[32], [34], from eight studies for DFS, [7], [8], [13], [29]–[32], [34] and from two studies for EFS [12], [31]. Because only two studies provided survival data for EFS, we did not perform sensitivity or subgroup analyses for EFS. The publication bias for EFS was also not detected because of the small sample size. HRs could be calculated from survival curves for three studies [12], [13], [32], from survival rates for two studies [33], [34], and from IPD-based analyses [9] for five studies [7], [8], [29]–[31].

**Table 1:** Study characteristics.

Study	No. of patients	Median age (range,years)	DNR/IDR ratio (mg/m^2^∶mg/m^2^)	Consolidation regimen		Median follow-up (years)	Included in analysis	HR estimation	Quality*
				DA	IA				
Berman *et al,* 1991	130	37.5 (17–60)	50×3∶12×3	DNR+Ara-C	IDR+Ara-C	2.5	CR, DFS, OS	HR	B1
Mandelli *et al,* 1991	249	62 (55–78)	45×3∶12×3	DNR+Ara-C+6-TG	IDR+Ara-C+6-TG	NR	CR, DFS, OS	HR	B1
Volger *et al,* 1992	230	60 (>15)	45×3∶12×3	DNR+Ara-C+6-TG	IDR+Ara-C+6-TG	NR	CR, DFS, OS	HR	B1
Wiernik *et al,* 1992	214	55 (>18)	45×3∶13×3	DNR+Ara-C	IDR+Ara-C	NR	CR, DFS, OS	HR	B1
Reiffers *et al,* 1996	220	NA (55–75)	50×3∶8×5	DNR+Ara-C	IDR+Ara-C	NR	CR, DFS, EFS, OS	HR	B1
Rowe *et al,* 2004	243	67 (56–86)	45×3∶12×3	Ara-C	Ara-C	NR	CR, DFS, OS	Surv. curves	B2
Gardin *et al,* 2007	416	72 (65–85)	45×4∶9×4	DNR/IDR+Ara-C	DNR/IDR+Ara-C	2.8	CR	Surv. rates	B1
Pautas *et al,* 2010	468	60 (50–70)	80×3∶12×3or4	Ara-C	Ara-C	4.1	CR, EFS, OS	Surv. curves	B1
Chevallier *et al,* 2010	823	48 (17–60)	60×3∶8×5	DNR+Ara-C	IDR+Ara-C	4.4	CR, DFS, OS	Surv. rates	B1
Ohtake *et al,* 2011	1057	47 (15–64)	50×5∶12×3	MIT+Ara-C,DNR+Ara-C, ACM+Ara-C, VP-16+VCR+VDS+Ara-C/Ara-C	MIT+Ara-C,DNR+Ara-C, ACM+Ara-C, VP-16+VCR+VDS+Ara-C/Ara-C	4	CR, DFS, OS	Surv. curves	A

IDR: idarubicin; DNR: daunorubicin; Ara-C: cytosine arabinoside; MIT: mitoxantrone; ACM: aclarubicin; VDS: vindesine; VP-16: etoposide; VCR: vincristine; 6-TG: 6-thioguanine; NA: not applicable.

* Quality: A, low risk of bias; B1, low-moderate risk of bias; B2, moderate-high risk of bias; C, high risk of bias.

The median age of the patients ranged from 37·5 to 72 years old. The assigned daunorubicin dose in the DA arm was 45–60 mg/m^2^ daily for 3 days in seven trials [7], [8], [29]–[32], [34], 45 mg/m^2^ daily for 4 days in one trial [33], 80 mg/m^2^ daily for 3 days in one trial [12], and 50 mg/m^2^ daily for 5 days in one trial [13]. The subjects in the IA arm were allocated idarubicin 12 mg/m^2^ daily for 3 days, 13 mg/m^2^ daily for 3 days, 9 mg/m^2^ daily for 4 days, 8 mg/m^2^ daily for 5 days, or 12 mg/m^2^ daily for 4 days. In our analysis, a total dose of daunorubicin greater than 180 mg/m^2^ was considered to be a high dose. The CR rate ranged from 40% to 83% in the IA arms and from 39% to 81% in the DA arms. Five trials had no reported median follow-up. Formal critical quality appraisal of the ten trials indicated that the risk of bias was low in one trial (quality A) [13], low to moderate in eight trials (quality B1) [7], [8], [29]–[31], [33], [34], and moderate to high in one trial (quality B2) [12].

### Meta-analysis

**Figure 2:**
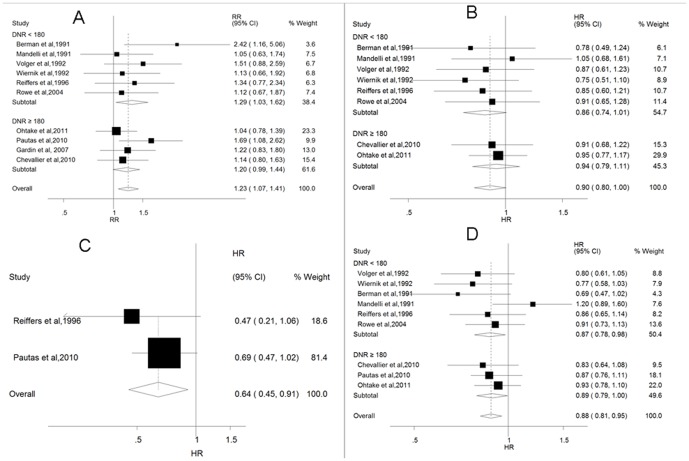
Forest plot of the RR/HR. The size of the squares reflects each study's relative weight, and the diamond (◊) represents the aggregate RR/HR and 95% CI. (A) Complete remission (*p* = 0.004); (B) Disease-free survival (*p* = 0.06); (C) Event-free survival (*p* = 0.013); (D) Overall survival (*p* = 0.002).

Patients randomly assigned to IA arms had significantly higher CRs than patients randomly assigned to receive DA chemotherapy (RR = 1·23; 95% CI = 1·07–1·41, *p* = 0.004; *p* = 0·541 for heterogeneity, Figure 2A). DFS was not significantly improved with IA compared to DA (HR = 0·90; 95% CI = 0·80–1·00, *p* = 0.06; *p* = 0·953 for heterogeneity, Figure 2B). A significant EFS (HR = 0·64; 95% CI = 0·45–0·91, *p* = 0.013; *p* = 0·402 for heterogeneity, Figure 2C) benefit of IA was documented. The difference in the overall survival was statistically significant (HR = 0·88; 95% CI = 0·81–0·95, *p* = 0.02; *p* = 0·450 for heterogeneity, Figure 2D), indicating a 12·0% decrease in hazard events in IA arms compared with DA arms.

### Sensitivity analysis

A single study included in the meta-analysis was deleted each time to reflect the influence of the individual data set on the pooled RRs/HRs, and the corresponding pooled results were not obviously materially altered (data not shown). However, I^2^ ranged from 2% to 40.6%, indicating that the heterogeneity was slightly materially altered.

### Subgroup analysis

As described in the protocol given in the Methods section, the studies were summarised in subgroups, according to a cut-off value regarding certain characteristics. The subgroups are shown in Table 2 according to patient and study characteristics. The subgroup analysis was performed according to a variety of criteria, and the outcomes are shown in Table 3. There were no significant differences for CR, DFS, or OS among older patients between the two groups. Patients who received idarubicin showed better overall survival (HR 0·89, 95% CI = 0·79–1·00, *P* = 0·042; *P* = 0·749 for heterogeneity) than patients who received high doses of daunorubicin, and the median age of these patients was younger than 60 years old.

**Table 2:** Subgroups according to patient and study characteristics.

Characteristics	Berman *et al,* 1991	Mandelli *et al,* 1991	Volger *et al,* 1992	Wiernik *et al,* 1992	Reiffers *et al,* 1996	Rowe *et al,* 2004	Gardin *et al,* 2007	Chevallier *et al,* 2010	Pautas *et al,* 2010	Ohtake *et al,* 2011
Publication before 2003	+	+	+	+	+	−	−	−	−	−
Median age >60 years old	−	+	−	−	NA	+	+	−	−	−
Total dose of DNR <180 mg/m^2^	+	+	+	+	+	+	−	−	−	−

DNR: daunorubicin; NA: not applicable.

**Table 3:** Subgroup analysis according to the characteristics.

Criteria		CR (RR)				DFS (HR)				OS (HR)			
		Fixed effects (95% CI)	Random effects (95% CI)	H *(p)*	I *(p)*	Fixed effects (95% CI)	Random effects (95% CI)	H *(p)*	I *(p)*	Fixed effects (95% CI)	Random effects (95% CI)	H *(p)*	I *(p)*
All		1·23 (1·07–1·41)	1·23 (1·07–1·41)	0.54	NA	0.90 (0.80–1·00)	0.90 (0.80–1·00)	0.473	NA	0·88 (0·81–0·95)	0.88 (0.81–0.95)	0.159	NA
Publication before 2003	+	1·34 (1·04–1·72)	1·34 (1·04–1·72)	0.41	0.426	0·85 (0·72–1·01)	0·85 (0·72–1·01)	0.28	0.453	0·86 (0·75–0.98)	0·86 (0·72–1·02)	0.03	0.674
	−	1·18 (1·00–1·40)	1·18 (1·00–1·40)	0.51		0·93 (0·80–1·08)	0·93 (0·80–1·08)	0.96		0·89 (0·81–0·99)	0·89 (0·81–0·99)	0.893	
Median age >60 years old	+	1·15 (0·88–1·49)	1·15 (0·88–1·49)	0.89	0.810	0·96 (0·74–1·25)	0·96 (0·74–1·25)	0.29	0.829	1·00 (0·84–1·20)	1·03 (0·78–1·34)	0.14	0.255
	−	1·25 (1·06–1·49)	1·30 (1·05–1·62)	0.20		0·89 (0·77–1·02)	0·89 (0·77–1·02)	0.609		0·85 (0·77–0·94)	0·85 (0·77–0·94)	0.267	
Total dose of DNR <180 mg/m^2^	+	1·29 (1·03–1·62)	1·29 (1·03–1·62)	0.50	0.576	0·86 (0·74–1·01)	0·86 (0·74–1·01)	0.344	0.493	0·87 (0·78–0.98)	0·87 (0·76–1·00)	0.047	0.848
	−	1·19 (1.00–1·42)	1·20 (0·99–1·44)	0.35		0·94 (0·79–1·11)	0·94 (0·79–1·11)	0.815		0·89 (0·79–1·00)	0·89 (0·79–1·00)	0.749	

H: Heterogeneity; I: Interaction; NA: not applicable.

### Publication bias

**Figure 3:**
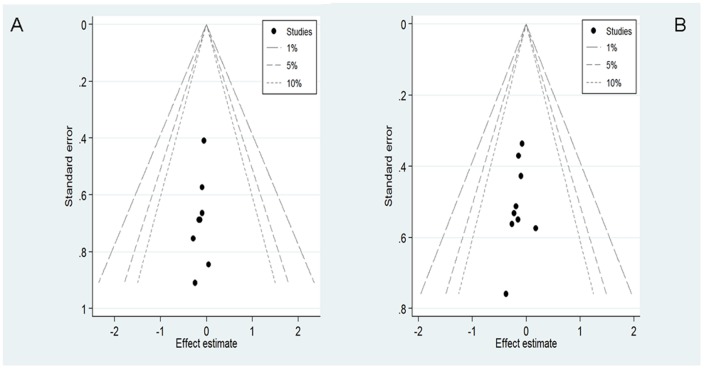
Contour-enhanced funnel plot for publication bias test. (A)Disease-free survival; (B) Overall survival.

Potential publication bias was estimated with the Begg-Mazumdar test and the Egger test. All of the studies investigating DFS yielded a Begg's test score of *p* = 0·083 and an Egger's test score of *p* = 0·238. Similar results were found for OS (*p* = 0·095 and 0·397, respectively). Contour-enhanced funnel plots (Figure 3, A and B) indicated that all studies were within the nonsignificant area, and no studies were in significant areas (i.e., from *P*<0.01 to *P*<0.05) for both HRs. It suggested that there was no publication bias for DFS and OS.

## Discussion

A prospective, randomised clinical trial is the accepted standard to compare different treatments, such as different anthracyclines, as induction regimens for newly diagnosed AML. The reported results of prospective, randomised, clinical trials were conflicting in the 1990s [7], [8], [29]–[31]. One meta-analysis reported better remission rates and better overall survival with idarubicin (12 to 13 mg/m^2^ for 3 days) than with daunorubicin (45 to 50 mg/m^2^ for 3 days) in combination with Ara-C. In fact, based on IPD analysis, no superior survival effects of idarubicin were detected in any prospective, randomised clinical trials in the 1990s [9]. Because of conflict over the utility of equipotent doses of daunorubicin, the results of the previous meta-analysis were renounced, and new recommendations from the NCCN and ELN continued to suggest that the choice of idarubicin or daunorubicin was of little consequence.

What is the truth about the optimal use of anthracyclines (daunorubicin or idarubicin) in induction treatment for AML? Our pooled results suggest that IA is associated with a significant advantage in CR (RR = 1·23; 95% CI = 1·07–1·41), EFS (HR = 0·64; 95% CI = 0·45–0·91), and OS (HR = 0·88; 95% CI = 0·81–0·95) but not in DFS (HR = 0·90; 95% CI = 0·80–1.00). Perhaps more patients died in remission, resulting in a non-significant benefit in DFS [9]. Our results were consistent with those of the prior meta-analysis, which was based on individual patient data (IPD) [9]. The same results were observed among younger patients, but no clinical benefits were documented in older patients. The extent to which the blast cells are cleared from the marrow in response to induction chemotherapy represents a clear indication of chemosensitivity or chemoresistance [37]. These observations have suggested that young patients might be more chemosensitive to idarubicin and that they should continue to be treated with idarubicin. However, our research did not simply rehash the previous research. We also expounded different outcomes of AML patients receiving equipotent dose of daunorubicin and idarubicin. Our results give the impression of idarubicin being superior in overall survival to daunorubicin in younger AML populations, although younger AML patients in control groups were given high doses of daunorubicin (no less than 180 mg/m^2^).

AML-related prognostic factors include age, white blood cell (WBC) count, the existence of a prior MDS, previous cytotoxic therapy for another disorder, and cytogenetic and molecular genetic changes in leukaemic cells at diagnosis [6]. We decided to examine whether these prognostic factors would influence the results. Finally, we only used the prognostic factor of a median age of 60 years old as a cut-off value to conduct subgroup analysis because the data for other prognostic factors were absent.

Endpoints might have differed among trials, especially the earlier and later ones, as some definitions of AML endpoints have changed since the Cheson criteria [38] were published. Thus, a publication start date of 2003 was used to perform subgroup analyses to determine whether the difference would influence the final results.

Whether equipotent doses were used in these randomised studies was frequently questioned. The cumulative anthracycline dose for induction has been suggested to be at least 180 mg/m^2^ of daunorubicin or 36 mg/m^2^ of idarubicin for young patients [6], [39]. On the basis of the NCCN and ELN recommendations, we used a total dose of 180 mg/m^2^ of daunorubicin as the cut-off value to complete the subgroup analyses. Although a significant CR was not observed, patients who received idarubicin showed better overall survival (HR 0·89, 95% CI = 0·79–1·00, *P* = 0·042) than those receiving at least 180 mg/m^2^ of daunorubicin, and the median age of these patients was younger than 60 years old. The optimal dose of daunorubicin is unknown. Daunorubicin dose intensification has been studied by several cooperative groups [40]–[42]. In young adults (60 years old or younger) with AML, an escalation of the dose (90 mg/m^2^ for 3 days) of daunorubicin to twice the conventional dose (45 mg/m^2^ for 3 days) improved both the CR rate and survival duration [40]–[41]. In older AML patients (60–65 years), similar results were confirmed [42]. All of the above studies indicated that the dose of daunorubicin would influence treatment effectiveness in young AML patients. On-going randomisation between DA and IA has been undertaken to demonstrate whether there are different clinical outcomes between idarubicin (12 mg/m^2^ for 3 days) and daunorubicin (90 mg/m^2^ for 3 days) (NCT01145846). The recommendations of the NCCN and ELN regarding induction regimens (at least 60 mg/m^2^ daunorubicin) for young patients should be used with greater caution. Furthermore, the future outcomes of randomised, clinical trials could be used to update our research and clarify the best choice of anthracycline, daunorubicin or idarubicin.

Several limitations should be considered when interpreting the results of our analysis. First, our results were based on unadjusted estimates, whereas a more precise analysis could have been conducted if the individual data were available, which would have allowed for adjustment according to other co-variables. Second, the analyses were based on abstracted data and not on IPD. Complete data sets were not available for all of the studies included in this meta-analysis. Some other endpoints could not be included, such as reasons for failure to achieve CR (i.e., induction death or resistant disease), relapse and death during the 1^st^ CR. We also could not identify the subgroups of patients who might have benefitted according to performance status, cytogenetic risk group, FAB classification, absences of splenomegaly and extramedullary disease, and so on. Our inability to address these points limited the value of the current research greatly. Third, publication bias is another major concern in all meta-analyses because studies reporting positive or significant findings are more likely to be published than those reporting non-significant results. It is primarily authors, not editors, who decide not to go to press [43]. In this study, there was no statistically significant evidence of possible publication bias using Begg's test, Egger's test or contour-enhanced funnel plots, which are likely to gain widespread acceptance to detect publication bias. In fact, if given sufficient time for unpublished studies (‘grey literature’) to pass through the pipeline and be published, the publication bias might have been much smaller than expected [43]. Fourth, the heterogeneity among the trials could be another limitation of our meta-analysis, although we applied both a random-effects model and a fixed-effects model to combine the data. The absence of a statistically significant difference in the metaregression analysis we used to examine heterogeneity might justify the analysis. This result indicates that using an overall estimation of the comparison of IA and DA could be appropriate. However, as the number of trials was limited, careful interpretation of the heterogeneity is necessary. Therefore, we must explicitly state that caution is highly advisable when interpreting the subgroup analyses.

In conclusion, our analysis indicates that IA might improve the overall survival duration of young patients with newly diagnosed AML compared to DA, which is different from the the recommendations of the NCCN and ELN. However, these results cannot be used as a guideline for AML treatment. We must take into account that other factors, such as consolidation therapy and stem cell transplantation, as well as the therapy adopted for relapsed patients, play pivotal roles. Further study is needed to determine whether specific subgroups of young AML patients will benefit from IA. Nevertheless, with appropriate caution, our results can be used in the development of new, empirically based research.

## Supporting Information

Table S1 PRISMA 2009 Checklist.
(PDF)

Text S1 Protocol.
(DOC)
