# Supplementary material for: Meta-Analysis of Randomised Clinical Trials Comparing Idarubicin + Cytarabine with Daunorubicin + Cytarabine as the Induction Chemotherapy in Patients with Newly Diagnosed Acute Myeloid Leukaemia
Source: PLoS One. 2013 Apr 5;8(4):e60699. doi: 10.1371/journal.pone.0060699 (PMC3622517; doi:10.1371/journal.pone.0060699)
Supplement: Text S1 — Protocol. (DOC) [file pone.0060699.s002.doc]

**Meta-analysis of randomised clinical trials comparing idarubicin + cytarabine with daunorubicin+ cytarabine as the induction chemotherapy in patients with newly diagnosed acute myeloid leukaemia (Protocol)**

**A B S T R A C T**

This is the protocol for a review and there is no abstract. The objectives are as follows:

The main objective of this review is to determine whether the use of idarubicin + cytarabine (IA) is more effective than the use of daunorubicin+ cytarabine (DA) as induction chemotherapy for patients with newly diagnosed acute myeloid leukaemia.The primary outcome of interest for our analysis was survival (disease-free survival, event-free survival and overall survival); the secondary endpoint was complete remission.

**B A C K G R O U N D**

**Description of the condition**

As of today, the management of AML remains a challenge for haematologists. The first goal of treatment is to achieve complete remission (CR), and further treatment is performed to prevent relapse. Much focus has been placed on increasing CR and reducing relapse and mortality to increase disease-free-survival (DFS), event-free survival (EFS), and overall survival (OS). Several trials have suggested the potential utility of cladribine or gemtuznmab-ozogamycin for remission induction therapy; however, the “3 + 7” protocol currently remains the standard remission induction therapy for AML.

**Description of the intervention**

The current recommendation for young AML patients from the National Comprehensive Cancer Network (NCCN), based on a literature review and on expert consensus, is three days of an anthracycline (e.g., daunorubicin at a dose of at least 60 mg/m^2^ or idarubicin at a dose of 12 mg/m^2^), and seven days of cytarabine (100-200 mg/m^2^ continuous infusion). For patients younger than 60 years old, the induction therapy generally consists of 3 days of an anthracycline (e.g., daunorubicin at 45-60 mg/m^2^ or, as an alternative, idarubicin at 12 mg/m^2^) and 7 days of cytarabine (100-200 mg/m^2^ continuous infusion) (V2·2011: available at [http://www.nccn.org](http://www.nccn.org/)). The European Leukaemia Net (ELN) [6] also provides similar recommendations for AML treatment. These recommendations suggest that the choice of an anthracycline (daunorubicin or idarubicin) is of little consequence, assuming that equipotent doses are administered.

**Why it is important to do this review**

Which is the optimal anthracycline to use in AML, daunorubicin or idarubicin? There have been many studies aimed at establishing an ideal induction therapy for AML, but most of them have failed to demonstrate the true superiority of IA over DA. On-going randomisation between DA and IA is being administered to demonstrate whether the choice of an anthracycline is appropriate according to the recommendations of the NCCN and ELN regarding induction regimen (NCT01145846: available at http://www.clinicaltrials.gov).

**O B J E C T I V E S**

The main objective of this review is to determine whether the use of idarubicin + cytarabine (IA) is more effective than the use of daunorubicin+ cytarabine (DA) as induction chemotherapy for patients with newly diagnosed acute myeloid leukaemia. The primary outcome of interest for our analysis was survival (disease-free survival, event-free survival and overall survival); the secondary endpoint was complete remission.

**M E T H O D S**

Criteria for considering studies for this review

**Types of studies**

We will accept only RCTs for this review and we will include both, full text and abstract publications.

**Types of participants**

We will include trials on patients with newly diagnosed acute myeloid leukaemia, and we will not apply any restriction on age, gender or ethnicity.

**Types of interventions**

The intervention is defined as follow:

●Idarubicin + cytarabine (IA) as induction chemotherapy for newly diagnosed AML patients.

The control interventions are defined as follow.

●Daunorubicin+ cytarabine (DA) as induction chemotherapy for patients with newly diagnosed AML.

**Types of outcome measures**

**Primary outcomes**

●Disease-free survival: DFS will be defined as the time interval from random treatment assignment/entry to the study to first progression or relapse, death from any cause or to last follow-up.

●Event-free survival: EFS will be defined as the time interval from random treatment assignment/entry to the study to CR achievement failure, first relapse, or death from any cause or to last follow-up.

●Overall survival: OS will be defined as the time interval from random treatment assignment/entry to the study to death from any cause or to last follow-up.

**Secondary outcomes**

●Complete remission: CR will be defined as less than 5% blasts in a normocellular

marrow and peripheral blood counts showing ≥1×10^9^/L neutrophils and≥100×10^9^/L platelets, without evidence of extramedullary leukemia.

**Electronic searches**

A computer-based search was performed of MEDLINE, EMBASE, the Cochrane-controlled trials registry, the Cochrane Library, and the Science Citation Index through March 2012. The search strategy included the medical subject headings of “Acute myeloid leukemia” and “anthracycline.” The reference lists were screened of all of the identified trials and of the comprehensive reviews in the field. The volumes of abstracts of the annual meetings of the American Society of Hematology (ASH), the European Haematology Association (EHA), and the American Society of Oncology (ASCO) were screened from 1995 to 2011. Prospective and on-going trials were identified by searching the following prospective trials registers: http://www.anzctr.org.au, http://www.clinicaltrials.gov, http://isrctn.org, http://www.trialregister.nl/trialreg/index.asp, http://www.umin.ac.jp/ctr.

**Data collection and analysis**

**Selection of studies**

After the first reviewof all titles and abstracts of the identified studies from the above sources, two review authors will independently reject all studies that are clearly ineligible. We will assess selected studies by using an eligibility form regarding study design and compliance with inclusion criteria. If there is any doubt, we will include full text analysis and discuss eligibility with both review authors to finalise a decision. We prefer to include studies rather than to lose relevant data. According to PRISMA,

we will use a flow chart to document the study selection process, showing the total numbers of retrieved references, the numbers of included and excluded studies.

**Data extraction and management**

Two review authors will independently extract data according to Chapter Seven of the Cochrane Handbook for Systematic Reviews of Interventions by using the standardised data extraction form with following items.

●General information: author, title, source, publication date, country, duplicate publications.

●Quality assessment: allocation concealment, blinding (participants, personnel, outcome assessors), incomplete outcome data, selective outcome reporting, other sources of bias.

●Study characteristics: trial design, aims, setting and dates, source of participants, inclusion/exclusion criteria, comparability of groups, subgroup analysis, statistical methods, power calculations, treatment cross-overs, compliance with assigned treatment, length of follow-up, time point of randomisation.

●Participant characteristics: age, gender, ethnicity, number of participants recruited/allocated/evaluated, participants lost to follow-up.

●Interventions: setting, type of (multi-agent) chemotherapy (intensity of regimen, number of cycles), duration of follow-up.

●Outcomes: disease-free survival, event-free survival, overall survival, and complete remission

**Assessment of Quality of included studies**

Quality assessment was based on the reporting of the study methods and results, namely randomisation, generation and concealment of treatment allocation, blinding, handling of withdrawals and dropouts, analysis by intention to treat, comparability of characteristics at baseline, treatment protocol being clearly described, outcome definition, and the extent of follow-up being clearly described. Study quality was coded as A (low risk of bias), B1 (low-moderate risk of bias), B2 (moderate-high risk of bias), or C (high risk of bias); as Liddle *et al.* commented , these codes are intended to be compatible with those of the Cochrane Collaboration (Cochrane Handbook, version 5.0.1, available at http://www.cochrane-handbook.org). We did not explicitly score the methodological quality of the included trials because the ad hoc quality assessment scores might have lacked demonstrated validity and the results might not have been associated with quality .

**Measures of treatment effect**

For binary outcomes, we will calculate risk ratios (RR) with 95% confidence intervals (CI) for each trial and for continuous outcomes. For time-to-event outcomes, we will extract hazard ratio (HR) from published data.

**Assessment of heterogeneity**

We will pool all results by applying meta-analysis using a fixed-effect model.We will assess heterogeneity of treatment effects between trials by using a CHI^2^- test with a significance level at P <0.1. We will use the I² statistic to quantify possible heterogeneity (I² > 30% moderate heterogeneity, I² > 75% considerable heterogeneity). We will explore the potential causes of heterogeneity by subgroup analysis.

**Assessment of reporting biases**

In meta-analyses with at least ten trials, we will explore potential publication by generating a funnel plot and statistically tested by using the methods of Egger et al. and Begg et al. We will consider P < 0.1 significant for the aim of this test.

**Data synthesis**

We will use the Cochrane statistical package Review Manager (RevMan) 5.1 and STATA 10.0 for analysis. One review author will input data into software and a second review author will check it for accuracy.

**Subgroup analysis and investigation of heterogeneity**

We performed the subgroup analysis, which was pre-planned according to the prepared protocol for this meta-analysis, by limiting the meta-analysis to studies using the following criteria: (a) time of publication, before or after 2003; (b) median age, older or younger than 60 years old; and (c) total dose of DNR, greater than or less than 180 mg/m^2^. Interaction tests were used to compare the differences between estimates from different subgroups .
